# Integrated Multi-Omics Analysis Explores the Protective Effects and Potential Mechanisms of *Pulsatilla chinensis* on Canine Antibiotic-Associated Diarrhea

**DOI:** 10.3390/biom16050650

**Published:** 2026-04-27

**Authors:** Zixuan Zhao, Jianfang Wang, Zhoufeng Wu, Lihua Ye, Jiahan Wang, Yihan Wang, Yuman Zhao, Hua Zhang, Chaochao Luo, Jinjin Tong

**Affiliations:** 1 Animal Science and Technology College, Beijing University of Agriculture, Beijing 102206, China; 202530321117@bua.edu.cn (Z.Z.);; 2 College of Veterinary Medicine, Beijing University of Agriculture, Beijing 102206, China; 3 College of Life Sciences, Shihezi University, Shihezi 832003, China

**Keywords:** canine, diarrhea, microbial community, molecular dynamics simulation, *Pulsatilla chinensis*

## Abstract

Diarrhea is a common gastrointestinal disorder in animals, often worsened by antibiotic use. *Pulsatilla chinensis* (PC) is traditionally used for gastrointestinal issues, but its bioactive constituents and mechanisms remain unclear. This study investigated the preventive effects of PC in a canine model of antibiotic-associated diarrhea using an integrated multi-omics approach. LC–MS identified key constituents of PC, including anemoside B4, berberine, stigmasterol, and quercetin. In silico analyses predicted that stigmasterol and quercetin target EGFR and AKT1, modulating inflammation and epithelial repair via PI3K–Akt and IL-17 signaling pathways. In vivo, treatment with PC significantly reduced serum pro-inflammatory cytokines such as TNF-α and IL-6 and elevated immune markers including IgG and IgA compared to the control group. Furthermore, 16S rRNA analysis revealed that PC restored gut microbial diversity, reflected by increased Sobs and Chao1 indices, enriched beneficial *Lactobacillus*, and decreased the abundance of inflammation-associated taxa such as *Proteobacteria*, *Desulfobacterota*, and *Escherichia-Shigella*. These findings suggest that PC suppresses inflammation and remodels the gut microbiome, providing a mechanistic basis for its use as an herbal alternative to antibiotics. Future studies should include fecal microbiota transplantation and targeted metabolomics to establish causality and optimize therapeutic strategies.

## 1. Introduction

Diarrhea is among the most prevalent gastrointestinal disorders in animals and remains a major threat to health and welfare, largely through its close links to intestinal inflammation, compromised barrier function, and disruption of gut microbial homeostasis [1,2]. Clinically, it manifests as increased defecation frequency and loose stools, but its pathophysiology extends beyond fluid loss to include mucosal injury and dysregulated immune responses driven, in part, by microbial imbalance [3]. The gut microbiota is now recognized as a key determinant of host physiological resilience, shaping nutrient metabolism, immune homeostasis, and epithelial integrity; accordingly, dysbiosis is increasingly implicated in gastrointestinal pathology and systemic immune perturbation [4,5]. Antibiotics are frequently used to control diarrheal disease, yet their broad-spectrum activity can further destabilize the intestinal ecosystem, predisposing animals to antibiotic-associated diarrhea and persistent dysbiosis while also accelerating antimicrobial resistance and raising residue-related concerns [6]. These constraints have prompted increasing interest in alternative approaches that alleviate diarrheal symptoms while preserving intestinal microbial balance and mucosal integrity.

Traditional herbal medicines have long been used to manage gastrointestinal disorders and are increasingly recognized for their potential to alleviate inflammation and support intestinal recovery while exerting fewer adverse ecological effects than conventional antibiotics. Among these, PC has been widely applied in veterinary practice for the treatment of diarrhea and inflammatory enteric conditions. However, in contrast to its established clinical use, the mechanisms by which PC influences host intestinal function, particularly through interactions with the gut microbiota, remain insufficiently characterized. This limitation is especially relevant in canine diarrhea, where intestinal recovery following antibiotic therapy is often inconsistent and recurrence is common, suggesting that restoration of microbial and mucosal homeostasis, rather than pathogen elimination alone, is critical for sustained resolution [7].

Phytochemical studies indicate that PC contains diverse bioactive constituents with reported anti-inflammatory and antimicrobial properties, yet the specific components responsible for its intestinal effects and their functional relationships with microbial communities, host immune responses, and epithelial repair processes remain largely undefined [8,9]. This gap reflects a broader challenge in traditional Chinese medicine, where therapeutic efficacy arises from complex mixtures acting on multiple biological targets, complicating mechanistic interpretation using conventional single-target frameworks [10]. Integrating metabolomics, network pharmacology, docking, and molecular dynamics simulations bridges chemical profiling and dynamic binding to systematically reveal the multi-component, multi-target, multi-pathway mechanisms of complex systems [11]. This approach thereby overcomes the limitations of conventional single-target frameworks. A clearer understanding of the chemical basis and microbiota-associated mechanisms underlying PC activity is therefore essential to support its rational development as a microbiome-oriented therapeutic strategy.

The present study investigated the preventive effects of PC using an integrated multi-omics framework in a canine model of antibiotic-associated diarrhea. LC–MS–based chemical profiling was performed to characterize its principal constituents, followed by network pharmacology and molecular simulations to predict molecular targets and pathways related to intestinal inflammation and epithelial repair. In vivo analyses were conducted to evaluate clinical outcomes and host immune responses, including circulating inflammatory cytokines and immunoglobulin levels. In parallel, 16S rRNA gene sequencing was applied to assess gut microbiota remodeling associated with PC administration. By integrating chemical, computational, physiological, and microbiological evidence, this study aimed to clarify the microbiota-associated mechanisms underlying PC activity and to provide a mechanistic basis for its microbiota-modulating application in veterinary medicine.

## 2. Materials and Methods

### 2.1. LC–MS Analysis

LC–MS analysis was conducted using a Thermo Scientific Q Exactive HF-X high-resolution mass spectrometer coupled with a Thermo Scientific Vanquish Flex ultra-high-performance liquid chromatography (UHPLC) system (Thermo Fisher Scientific, Waltham, MA, USA). Data acquisition was performed using Xcalibur software (version 4.7), and data processing was carried out with MS-DIAL (version 4.9.221218) [12]. The relative abundance of the identified compounds was estimated based on the peak area integration from the total ion chromatograms (TICs). The constituents were ranked by their peak areas to identify the major components, representing a semi-quantitative profile.

Chromatographic separation was achieved on an ACQUITY UPLC HSS T3 column (Waters Corporation, Milford, MA, USA; 2.1 × 100 mm, 1.8 μm) at a flow rate of 0.40 mL/min. Mobile phase A consisted of water containing 0.1% formic acid, and mobile phase B was acetonitrile containing 0.1% formic acid. The gradient elution program was as follows: 5% B for 0.00–1.00 min, a linear increase from 5% to 95% B over 1.00–4.70 min, holding at 95% B for 4.70–6.00 min, a linear decrease to 5% B over 6.00–6.10 min, and equilibration at 5% B for 6.10–8.50 min. The autosampler temperature was maintained at 8 °C, the column temperature at 40 °C, and the injection volume was 2 μL.

Mass spectrometry was performed in both positive and negative heated electrospray ionization (HESI) modes. Source parameters were set as follows: spray voltage, 3.5 kV (positive) and 2.8 kV (negative); capillary temperature, 320 °C; auxiliary gas heater temperature, 300 °C; sheath gas flow rate, 40 arb; and auxiliary gas flow rate, 10 arb. Full MS scans were acquired at a resolution of 60,000 (FWHM) over an m/z range of 70–1000, with the AGC target set to standard and a maximum injection time of 100 ms. For data-dependent acquisition (DDA), the top 10 precursor ions were selected for MS/MS fragmentation at a resolution of 15,000 using a normalized collision energy (NCE) of 30%, with a dynamic exclusion time of 4 s. Quality control (QC) samples were injected at regular intervals throughout the analytical sequence to monitor system stability.

### 2.2. Preparation of PC Extract

The raw herbal material of *Pulsatilla chinensis* was purchased from Beijing Tongrentang Group Co., Ltd. (Beijing, China), and authenticated according to the standard procedures described in the Chinese Pharmacopoeia (2020 Edition). The *Pulsatilla chinensis* extract was prepared by refluxing the dried roots in 80% ethanol at 70 °C for 3 h, repeated three times. The filtrate was concentrated and further fractionated to obtain the n-butanol extract. The final yield of the extract was recorded, and the powder was stored at 4 °C until use. To ensure the reproducibility of the extraction process and batch-to-batch consistency, a standardized extraction procedure was strictly followed. The quality stability of the PC extract was further verified by LC-MS analysis, which confirmed a consistent chemical profile of major bioactive constituents, such as anemoside B4 and berberine, across different preparation batches.

### 2.3. Network Pharmacology Analysis

Canonical SMILES strings of the active compounds in PC were obtained from the PubChem database (https://pubchem.ncbi.nlm.nih.gov/, accessed on 22 September 2025). Potential targets of these compounds were predicted using SwissTargetPrediction (http://swisstargetprediction.ch/, accessed on 22 September 2025) [13]. Diarrhea-related targets were collected from the GeneCards (https://www.genecards.org/, accessed on 22 September 2025) [14], OMIM (https://omim.org/, accessed on 22 September 2025) [15], and DisGeNET (https://www.disgenet.org/, accessed on 22 September 2025) databases [16] using “diarrhea” as the search keyword. All disease-related targets were merged, and duplicate entries were removed. Target names were subsequently standardized to Gene Symbols using the UniProt database (https://www.uniprot.org, accessed on 22 September 2025).

The overlapping targets between PC-associated targets and diarrhea-related targets were identified and visualized using Venny 2.1 (v2.1; https://bioinfogp.cnb.csic.es/tools/venny/index.html, accessed on 22 September 2025). These intersecting targets were imported into the STRING database to construct a protein–protein interaction (PPI) network, with the confidence score threshold set at >0.4. The resulting network was visualized using Cytoscape software (version 3.9.1) [17], where isolated nodes were removed. Topological parameters, including degree, betweenness centrality, and closeness centrality, were calculated, and the top 10 hub genes were identified using the Degree, EPC, MNC, and MCC algorithms implemented in the CytoHubba plugin [18]. GO and KEGG pathway enrichment analyses were performed using the Metascape platform [19], with a significance threshold of *p* < 0.05. The top five enriched terms for BP, CC, and MF categories were selected. Thirteen key KEGG pathways were further identified after excluding human disease-related pathways. These data were integrated into Cytoscape to construct a drug–disease–target–pathway network, illustrating the potential regulatory pathways associated with PC in diarrhea.

### 2.4. Molecular Docking and Molecular Dynamics Simulation

The top six core targets were selected based on descending degree values derived from the PPI network. Chemical structures of the corresponding active compounds were retrieved from the PubChem database, and crystal structures of the target proteins were obtained from the RCSB Protein Data Bank (PDB). Protein structures were preprocessed using PyMOL (v2.5) to remove water molecules and co-crystallized ligands. Molecular docking was performed using AutoDock Vina (v1.1.2) to evaluate binding affinities between active compounds and target proteins, and docking conformations were visualized using PyMOL [20]. To further assess the stability and dynamic behavior of the ligand–protein complexes, molecular dynamics (MD) simulations were conducted for 100 ns using the GROMACS 2022 software package.

### 2.5. Animals and Experimental Design

All animal procedures were approved by the Animal Ethics Committee of Capital Medical University (approval no. BUA812403014) and conducted in accordance with the National Institutes of Health guidelines. Sixteen one-year-old Beagle dogs (8 males and 8 females, mean baseline body weight: 10.5 ± 1.2 kg) were obtained from Beijing Marshall Biotechnology Co., Ltd. (Beijing, China) and housed at the Experimental Animal Center of Beijing University of Agriculture under controlled environmental conditions (temperature, 20 ± 2 °C; relative humidity, 60–70%; 12 h light/dark cycle). Animals had free access to food and water during a 2-week acclimatization period. After acclimatization, dogs were assigned to two groups (*n* = 8 per group): a model control group (CON) and a PC treatment group (TRT). The random sequence was generated using computer-generated block randomization (block size 4–6) via SAS 9.4, stratified by sex and baseline body weight. Allocation concealment was strictly ensured using sequentially numbered, sealed, opaque envelopes (SNOSE) prepared by a statistician not involved in the experiment. Envelopes were opened only after each dog was enrolled and baseline data recorded. An independent researcher, unrelated to animal housing or treatment, performed the allocation immediately before the intervention initiation. Randomization occurred immediately before intervention initiation.

To minimize the influence of individual physiological and microbiota variations among the Beagle dogs, data collected on day 0 (pre-treatment) served as the internal healthy baseline for each subject. This self-controlled longitudinal design allowed for a more precise evaluation of the shifts from a healthy state to antibiotic-induced dysbiosis and the subsequent protective response. Furthermore, this streamlined design ensured strict adherence to the 3Rs (Replacement, Reduction, and Refinement) ethical principles by minimizing the total number of experimental companion animals required, avoiding the doubling of sample size that parallel healthy or PC-only groups would necessitate. The dosage of PC extract was set at 200 mg/kg based on previous pharmacological studies [21] and our preliminary pre-experiments. Prior to daily administration, the PC extract powder was resuspended in 0.9% (*w*/*v*) NaCl solution to achieve the required concentration and vortexed thoroughly to ensure a homogeneous suspension. To establish the antibiotic-associated diarrhea model, dogs in both groups received a daily intragastric gavage of enrofloxacin (10 mg/kg) and metronidazole (25 mg/kg) from day 1 to day 14. This specific enrofloxacin/metronidazole combination has been extensively validated as an AAD-inducing regimen [22]. Because healthy Beagle dogs possess a highly adaptable gut microbiota, a rigorous 14-day challenge at the upper end of the standard therapeutic dose range is necessary to reliably overcome intrinsic compensatory mechanisms. The enrofloxacin dosage (10 mg/kg/day) falls within the established canine therapeutic range (5–20 mg/kg/day), and the metronidazole dosage of 25 mg/kg/day is consistent with standard canine doses (15–25 mg/kg/day) [23].

Simultaneously, the TRT group was orally administered the PC extract (200 mg/kg) daily, while the CON group received an equivalent volume of 0.9% (*w*/*v*) NaCl solution. Clinical parameters and fecal characteristics were recorded to monitor the progression of diarrhea and the preventive efficacy of PC.

### 2.6. Sample Collection and Diarrhea Assessment

Clinical parameters, including body weight, heart rate, and rectal temperature, were recorded alongside fecal characteristics (pH, morphology, and diarrhea status) on days 0 and 14. Diarrhea severity was evaluated based on fecal output within 120 min, fecal water content, and stool consistency. Stool consistency was scored using the 7-point Bristol Stool Scale [24], in which scores of 1–2 indicate hard stools, 3–4 represent normal stools, and 5–7 indicate loose or watery stools characteristic of diarrhea. Fresh fecal samples were collected on either days 0 or 14 and immediately stored for subsequent gut microbiota analysis by 16S rRNA gene sequencing.

### 2.7. Biochemical Analyses of Serum

Blood samples were centrifuged at 9500× *g* for 10 min to isolate serum. Serum concentrations of IL-6, TNF-α, IgG, and IgA were measured using commercial enzyme-linked immunosorbent assay (ELISA) kits (Beijing Huaying Biological Co., Ltd., Beijing, China).

### 2.8. 16S rRNA Gene Sequencing and Bioinformatics Analysis

Genomic DNA was extracted from fecal samples using the Fast Pure Stool DNA Isolation Kit (MJYH, Shanghai, China, T10-100) according to the manufacturer’s instructions. DNA concentration and purity were assessed using a NanoDrop ND-2000 spectrophotometer (Thermo Fisher Scientific, Wilmington, DE, USA). The V3–V4 regions of the bacterial 16S rRNA gene were amplified using primers 341F (5′-CCTACGGGNGGCWGCAG-3′) and 805R (5′-GACTACHVGGGTATCTAATCC-3′). Purified PCR products were sequenced on an Illumina NextSeq 2000 PE300 platform (Illumina, San Diego, CA, USA). After quality filtering and trimming, an average of 73,332 optimized reads per sample were obtained, with the sequencing depth ranging from 60,065 to 79,656 reads across the samples. Raw sequencing data have been deposited in the NCBI Sequence Read Archive (accession no. PRJNA1091895). Bioinformatics analyses were performed using the Majorbio Cloud Platform. Taxonomic assignment of the representative sequences was performed using the SILVA database (Release 138) with a confidence threshold of 0.7. To ensure comparability across samples, the sequence data were rarefied to the lowest sequencing depth across all samples prior to alpha and beta diversity analyses. Alpha diversity indices, including Observed OTUs and Chao1, were calculated using Mothur (version 1.30.2) [25]. Beta diversity was assessed by principal coordinate analysis (PCoA) based on Bray–Curtis dissimilarity using the vegan package in R (version 2.4.3) [26], and statistical significance was evaluated by PERMANOVA. Differentially abundant taxa from phylum to genus levels were identified using linear discriminant analysis effect size (LEfSe), with thresholds set at an LDA score >2.5 and *p* < 0.05.

### 2.9. Statistical Analysis

All statistical analyses were performed using SPSS software (version 26.0; IBM Corp., Armonk, NY, USA). Data are presented as mean ± standard deviation (SD). Intra-group differences were analyzed using one-way analysis of variance (ANOVA), while comparisons between two groups were conducted using Student’s *t*-test. For data that were not normally distributed, such as specific alpha-diversity indices and microbial relative abundances, the non-parametric Wilcoxon rank-sum test was used. For differential abundance analyses of the microbiota, including the Wilcoxon rank-sum test and LEfSe, *p*-values were adjusted for multiple testing using the false discovery rate (FDR) method to minimize Type I errors. A *p* < 0.05 was considered statistically significant.

## 3. Results

### 3.1. LC–MS Profiling of Chemical Constituents in Pulsatilla chinensis Extract

**Figure 1:**
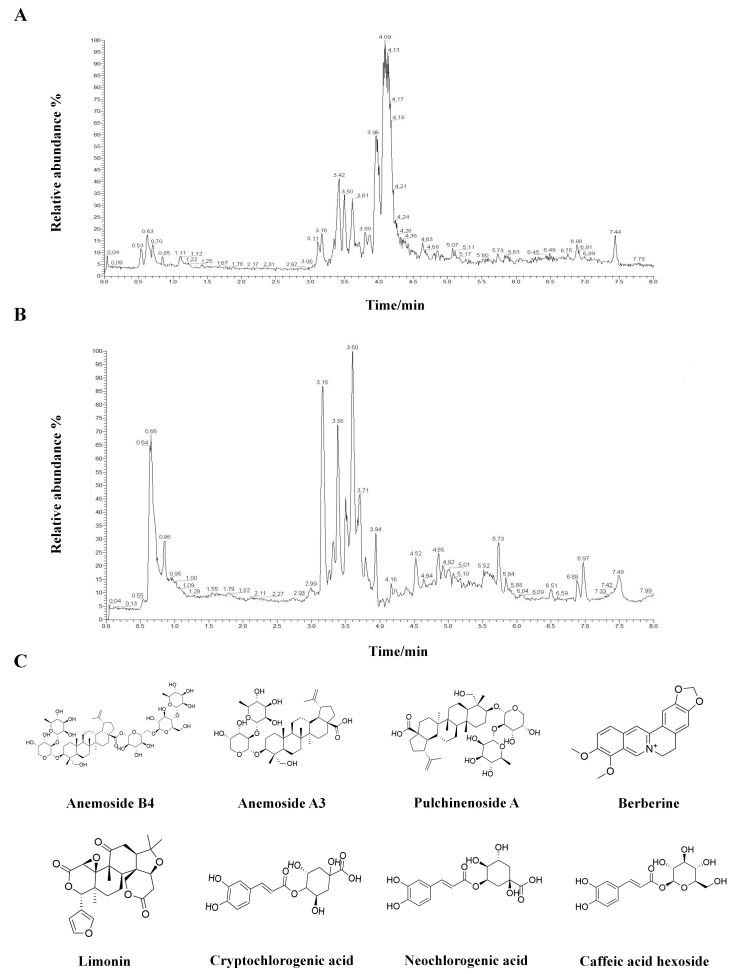
Qualitative analysis of the chemical constituents in PC extract using liquid chromatography-tandem mass spectrometry (LC-MS/MS). (**A**,**B**) Total ion chromatograms (TICs) of the PC extract acquired in positive (**A**) and negative (**B**) ion modes. (**C**) Chemical structures of representative bioactive components identified in the extract. Green lines indicate the integration baselines used for peak area calculation.

Total ion chromatograms acquired in both positive and negative ionization modes showed good peak resolution and stable signal responses, indicating effective chromatographic separation of the chemical constituents (Figure 1A,B). Based on high-resolution mass spectrometric data combined with database matching, a total of 2663 metabolites were identified, including 1525 compounds detected in positive ion mode and 1138 compounds detected in negative ion mode. Based on the established screening criteria regarding relative abundance, pharmacokinetic properties, and reported bioactivity, 20 core active constituents were carried forward into the in silico analysis. The identified metabolites covered a wide range of chemical classes. Triterpenoid saponins, represented primarily by anemosides, and alkaloids, such as berberine, constituted the dominant bioactive categories, followed by phytosterols, flavonoids, and phenolic acids.

Using UPLC–Q Exactive MS/MS analysis, the chemical composition of the PC extract was systematically characterized. Representative chemical structures of the major bioactive constituents are shown in Figure 1C. Relative abundance analysis based on peak area integration and mass spectral response revealed distinct ionization patterns across modes. In the positive ion mode, berberine exhibited the highest signal intensity, followed by limonin and quercetin. In the negative ion mode, anemoside B4, identified by characteristic fragment ions, showed prominent abundance, along with phenolic acids including cryptochlorogenic acid and chlorogenic acid. Detailed information on representative compounds with reported biological relevance, including anemoside B4, berberine, quercetin, stigmasterol, limonin, and cryptochlorogenic acid, is indicated in Table 1. Collectively, these results indicate that PC extract is enriched in saponins, alkaloids, and sterols, providing a chemical basis for its subsequent pharmacological evaluation.

### 3.2. Network Pharmacology Analysis of Pulsatilla chinensis in Diarrhea Intervention

**Figure 2:**
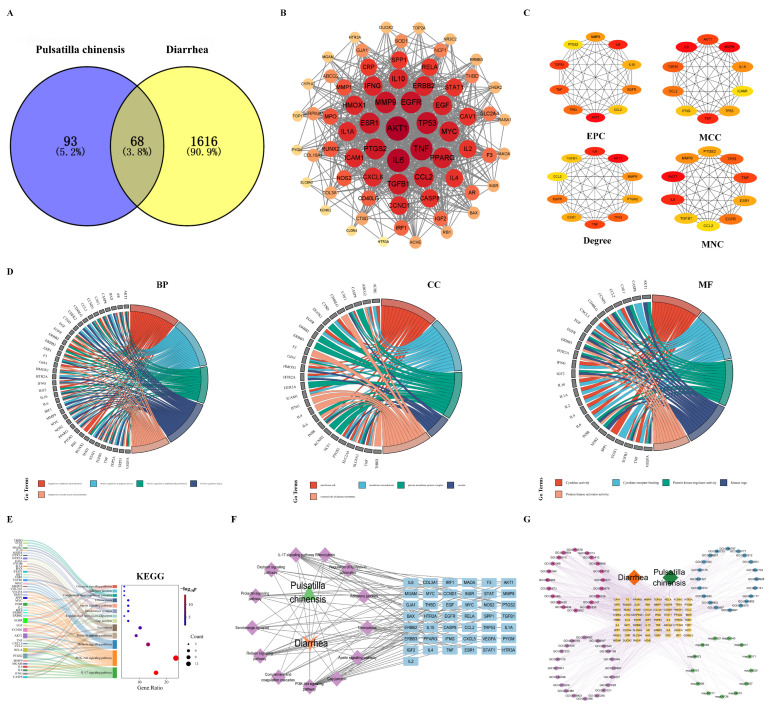
Network pharmacology analysis of potential therapeutic targets and molecular mechanisms of PC against diarrhea. (**A**) Venn diagram of PC active ingredients and diarrhea common targets. (**B**) PPI network of PC targets against diarrhea. (**C**) The top ranked hub genes in the PPI network as determined by CytoHubba. The node color gradient from yellow to red indicates an increasing degree value. (**D**) GO analysis of the 68 overlapping genes. (**E**) KEGG pathway analysis of the 68 overlapping genes. (**F**) Component–target–pathway network of PC in the treatment of diarrhea. The green triangle represents Pulsatilla chinensis, the pink/purple diamonds represent the disease and signaling pathways, and the blue rectangles represent the target genes. (**G**) Construction of the interaction network for the top 20 GO annotations, top 20 KEGG pathways, and the overlapping genes. The orange diamond represents the disease (Diarrhea), and the dark green diamond represents the herb (*Pulsatilla chinensis*). The central yellow rectangles represent the overlapping target genes. The surrounding pink, purple, and blue circles denote the top enriched Gene Ontology (GO) terms, while the light green circles indicate the enriched KEGG pathways.

To explore the potential molecular mechanisms underlying the antidiarrheal effects of PC, a network pharmacology strategy was applied. A total of 161 putative targets associated with the identified active ingredients were obtained from the SwissADME and TCMSP databases. In parallel, 1684 diarrhea-related targets were collected from the GeneCards, OMIM, and DisGeNET databases. Intersection analysis identified 68 shared targets between PC-associated targets and diarrhea-related targets (Figure 2A). These 68 overlapping targets were imported into Cytoscape 3.9.1 to construct a protein–protein interaction (PPI) network (Figure 2B). Network topology analysis revealed several highly connected nodes, including *AKT1*, *IL6*, *TNF*, *TP53*, and *EGFR*, suggesting their potential involvement in the protective effects of PC. Further hub gene screening using the CytoHubba plugin, based on four algorithms (Degree, EPC, MNC, and MCC), identified the top 10 hub genes within the network (Figure 2C).

GO enrichment analysis of the overlapping targets is shown in Figure 2D. In the Biological Process (BP) category, targets were mainly enriched in regulation of apoptotic processes, epithelial cell proliferation, and smooth muscle cell proliferation. Cellular Component (CC) analysis showed significant enrichment in membrane rafts, membrane microdomains, plasma membrane protein complexes, caveolae, and the external side of the plasma membrane. For Molecular Function (MF), the predominant terms included cytokine activity, cytokine receptor binding, protein kinase regulator activity, and protein kinase activator activity.

KEGG pathway enrichment analysis identified 13 significantly enriched signaling pathways (Figure 2E), among which the PI3K–Akt signaling pathway, IL-17 signaling pathway, and relaxin signaling pathway were the most prominent. To further visualize the relationships among active components, targets, and pathways, a component–target–pathway network was constructed using Cytoscape (Figure 2F), demonstrating that PC potentially regulates 43 core targets across 13 key signaling pathways. In addition, an integrated interaction network was generated to illustrate the associations among the top enriched BP, CC, MF terms, and KEGG pathways (Figure 2G), highlighting the network-based regulatory features of PC in diarrhea-related biological processes.

### 3.3. Molecular Docking and Molecular Dynamics Simulation Analysis

**Figure 3:**
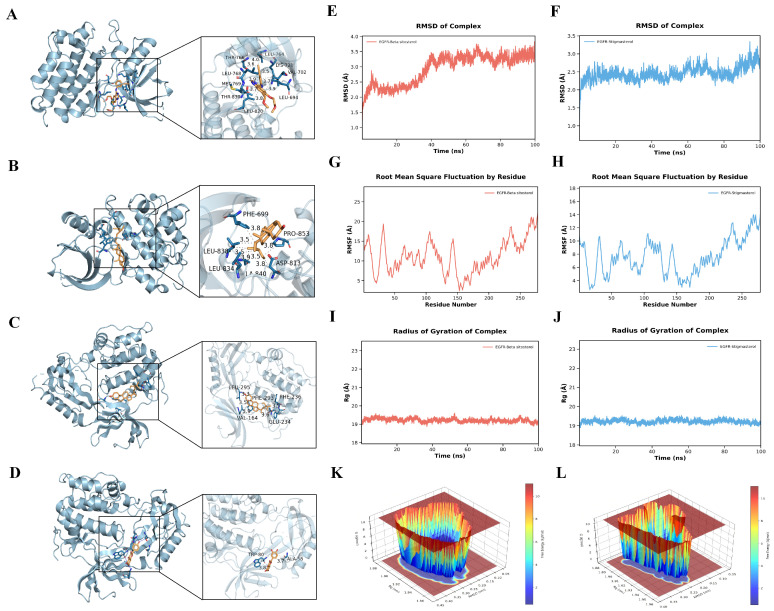
Molecular docking and molecular dynamics simulation analysis. Three-dimensional mapping of binding modes between active ingredients and core targets: (**A**) Stigmasterol–*AKT1*, (**B**) Stigmasterol–*EGFR*, (**C**) Quercetin–*AKT1*, and (**D**) Quercetin–*EGFR*. RMSD of the protein–ligand complexes: (**E**) *EGFR*–Stigmasterol and (**F**) *EGFR*–Quercetin. RMSF showing the flexibility of individual amino acid residues: (**G**) *EGFR*–Stigmasterol and (**H**) *EGFR*–Quercetin. Radius of Gyration (Rg) assessing the structural compactness: (**I**) *EGFR*–Stigmasterol and (**J**) *EGFR*–Quercetin. Gibbs free energy landscape of the protein–ligand complexes: (**K**) *EGFR*–Stigmasterol and (**L**) *EGFR*–Quercetin.

To evaluate the binding potential between core bioactive constituents of PC and key therapeutic targets, molecular docking was performed using representative active compounds, including anemoside B4, anemoside A3, pulchinenoside A, berberine, stigmasterol, and quercetin, against six core targets (*AKT1*, *EGFR*, *IL6*, *TP53*, *TNF*, and *MMP9*). Docking results are summarized in Table 2. All ligand–target pairs exhibited binding energies lower than −5.0 kcal/mol, indicating favorable binding affinities and the formation of stable interaction conformations. Among the tested compounds, stigmasterol and quercetin displayed comparatively stronger binding activities, particularly showing the lowest binding energies in their interactions with *AKT1* and *EGFR*. Based on binding energy ranking and interaction conformations, the four most favorable target–compound complexes were selected for three-dimensional visualization using PyMOL (Figure 3A–D).

Based on the molecular docking results, stigmasterol and quercetin exhibited the lowest binding energies among all identified candidates, indicating the highest theoretical binding affinities with the core targets. Consequently, to further evaluate the interaction stability and dynamic behavior of the most potent ligand–protein complexes, MD simulations were conducted for the Stigmasterol–*EGFR* and Quercetin–*EGFR* complexes for 100 ns. Root mean square deviation analysis showed that both systems reached stable plateaus during the middle to late stages of the 100 ns simulation (Figure 3E,F), indicating conformational stabilization. Residue-level flexibility was assessed using root mean square fluctuation (RMSF) analysis. The RMSF profiles demonstrated relatively low fluctuations of amino acid residues surrounding the ligand-binding regions in both complexes, suggesting limited local structural perturbation upon ligand binding (Figure 3G,H). In addition, the radius of gyration (Rg), which reflects the compactness of the protein structure, remained stable throughout the simulation, indicating that *EGFR* maintained its structural integrity without significant unfolding or expansion (Figure 3I,J).

To further characterize the conformational stability and dominant binding states of the complexes, free energy landscape (FEL) analyses were constructed based on RMSD and radius of gyration values (Figure 3K,L). In the FEL plots, color gradients from blue to red represent transitions from low to high free energy states, with deep blue regions indicating the most energetically favorable conformations. Both complexes displayed well-defined and concentrated low-energy basins, suggesting the presence of stable and highly probable binding conformations for stigmasterol–*EGFR* and quercetin–*EGFR* complexes.

### 3.4. Effects of PC Treatment on Physiological Indicators and Clinical Symptoms

**Figure 4:**
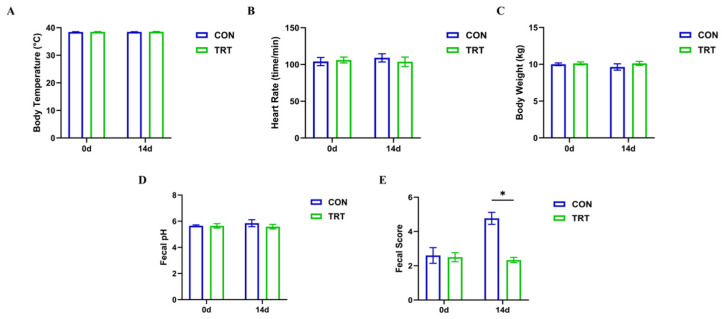
PC ameliorates clinical symptoms, suppresses systemic inflammation, and enhances immune function in diarrheal dogs. (**A**–**D**) General physiological indicators including (**A**) Body temperature, (**B**) Heart rate, (**C**) Body weight, and (**D**) Fecal pH. No significant differences were observed, indicating the safety of PC. (**E**) Fecal scores evaluating diarrhea severity. Data are presented as mean ± SD (*n* = 8). * *p* < 0.05 vs. CON group (day 14).

To evaluate the protective effects of PC treatment, physiological parameters, clinical symptoms, and serum immunological indicators were monitored in dogs with antibiotic-associated diarrhea. General physiological indices, including body temperature (Figure 4A), heart rate (Figure 4B), body weight (Figure 4C), and fecal pH (Figure 4D), were recorded on days 0 and 14. No significant differences were observed between the CON and TRT groups at either time point (*p* > 0.05), indicating that PC administration did not induce detectable alterations in basic physiological functions during the experimental period. After 7 days of antibiotic administration, all dogs exhibited clinical signs consistent with antibiotic-associated diarrhea, including lethargy, perianal erythema, and loose stools. Compared with baseline values on day 0 (2.60 ± 0.46), diarrhea scores in the CON group increased significantly by day 14 (4.83 ± 0.45), confirming successful establishment of the diarrhea model (Figure 4E).

### 3.5. PC Attenuates Serum Pro-Inflammatory Cytokines and Modulates Immunoglobulin Levels

**Figure 5:**
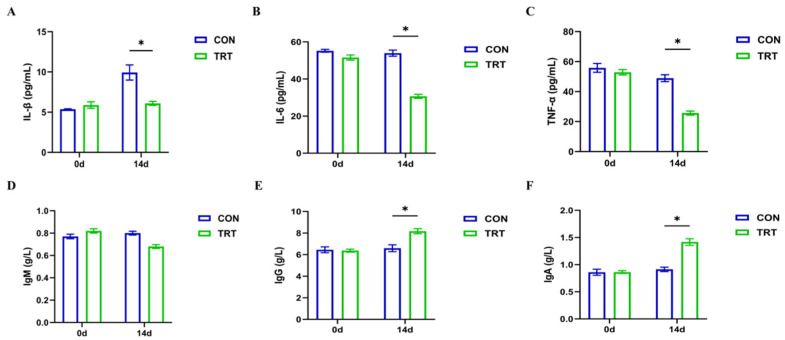
PC regulates inflammatory responses and immune function. (**A**–**C**) Serum cytokine concentrations at 0d and 14d, including (**A**) IL-1β, (**B**) IL-6, and (**C**) TNF-α. (**D**–**F**) Serum immunoglobulin levels at 0d and 14d, including (**D**) IgM, (**E**) IgG, and (**F**) IgA. Data are presented as mean ± SD (*n* = 8). * *p* < 0.05 vs. CON group (14d).

To evaluate the anti-inflammatory and immunomodulatory effects of PC, we analyzed serum cytokine and immunoglobulin levels on days 0 and 14 (Figure 5). On day 0, there were no significant differences in the measured indices between the CON and TRT groups. However, by day 14, distinct differences were observed. As shown in Figure 5A–C, serum concentrations of pro-inflammatory cytokines, including IL-1β (Figure 5A), IL-6 (Figure 5B), and TNF-α (Figure 5C), were significantly lower in the TRT group compared with the CON group (*p* < 0.05). This suggests that PC treatment effectively suppressed the systemic inflammatory response induced in the model. In terms of humoral immunity, PC treatment exhibited differential regulatory effects (Figure 5D–F). The serum levels of IgG (Figure 5E) and IgA (Figure 5F) in the TRT group were significantly increased compared with the CON group on day 14 (*p* < 0.05), indicating an enhancement of immune defense capabilities. Interestingly, the level of IgM (Figure 5D) in the TRT group was significantly lower than that in the CON group on day 14 (*p* < 0.05). Collectively, these results demonstrate that PC exerts a protective effect by inhibiting excessive inflammation and modulating immunoglobulin production.

### 3.6. Assessment of Gut Microbial Alpha-Diversity

**Figure 6:**
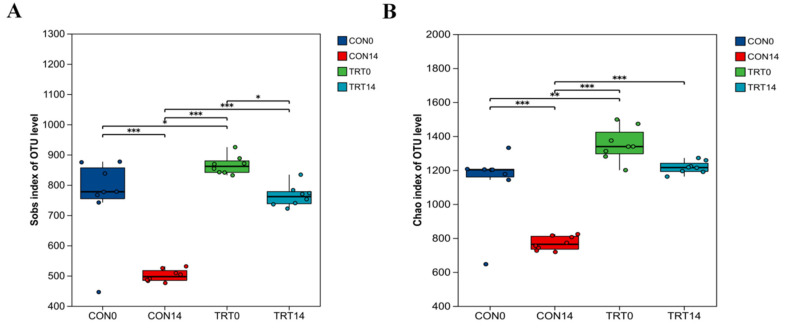
PC altered the Alpha diversity indices of gut microbiota in diarrheal dogs. (**A**) Sobs index. (**B**) Chao1 index. Data are presented as box plots showing the median and interquartile range (*n* = 8). Statistical significance was determined using the Wilcoxon rank-sum test: * *p* < 0.05, ** *p* < 0.01, *** *p* < 0.001.

To evaluate the protective effect of PC on gut microbiome stability, we analyzed the alpha diversity of fecal samples, specifically focusing on species richness indices Sobs and Chao1 as illustrated in Figure 6. At day 0, no significant differences were observed between the CON and TRT groups (*p* > 0.05), indicating a consistent initial microbial community structure. Following 14 days of antibiotic administration, the gut microbiota of the CON group exhibited severe dysbiosis, where species richness was dramatically depleted as evidenced by significantly lower Sobs and Chao1 indices in the CON14 group compared to the baseline (*p* < 0.001). In contrast, PC administration effectively attenuated this antibiotic-induced loss of microbial richness, as the Sobs and Chao1 indices in the TRT14 group were significantly higher than those in the CON14 group (*p* < 0.05).

### 3.7. Pulsatilla chinensis Modulates Gut Microbiota Structure and Composition

**Figure 7:**
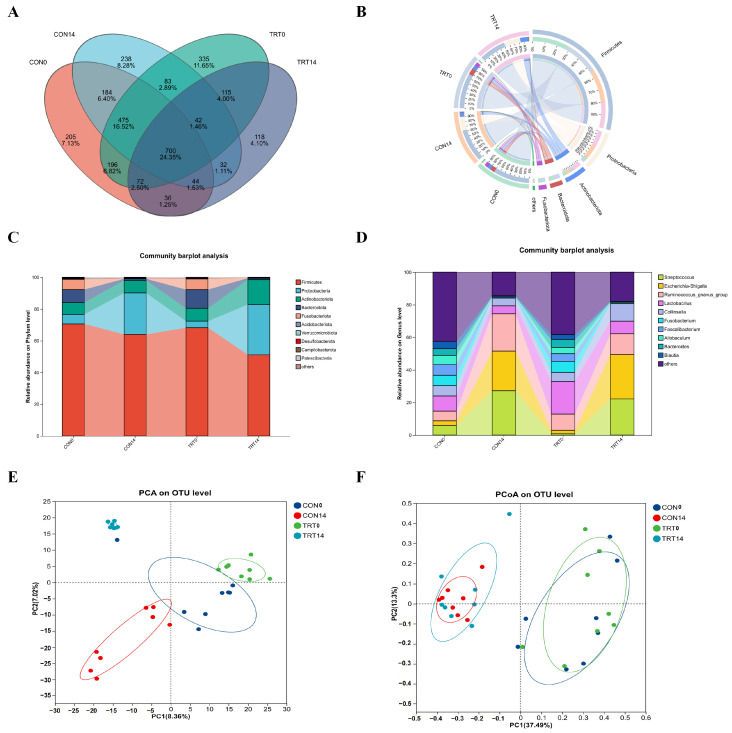
PC regulated the intestinal microflora and modulated the microbial community structure in diarrheal dogs. (**A**) Venn diagram of OTUs. Different colors represent different groups. (**B**) Circos diagram at phylum level. (**C**) Relative abundance plots displaying the differences in the general microbial community. (**D**) Relative abundance plots displaying the differences in the general microbial community at the genus level. (**E**) PCA diagram for CON and TRT groups. (**F**) PCoA diagram for CON and TRT groups.

To characterize the effects of PC treatment on gut microbial community structure in diarrheal dogs, fecal samples were subjected to 16S rRNA gene sequencing. Venn diagram analysis based on operational taxonomic units (OTUs) revealed that 700 OTUs were shared among all experimental groups, indicating the presence of a common core microbiota (Figure 7A). At the phylum level, Firmicutes, Proteobacteria, and Actinobacteriota constituted the dominant bacterial taxa across all samples (Figure 7B,C). Compared with the CON group, the TRT group exhibited altered relative abundances of several dominant phyla, showing a shift in microbial composition following PC administration. At the genus level, community composition analysis demonstrated that PC treatment was associated with changes in the relative abundance of several key genera, including *Streptococcus*, *Escherichia–Shigella*, and *Lactobacillus* (Figure 7D). Notably, genera commonly associated with opportunistic pathogens showed reduced relative abundance in the TRT group, whereas probiotic-related genera displayed an increasing trend. Multivariate analyses further revealed differences in overall microbial community structure between groups. Principal component analysis (PCA) and principal coordinate analysis (PCoA) based on Bray–Curtis dissimilarity demonstrated distinct clustering patterns among samples (Figure 7E,F). By day 14, the microbial community structure of the TRT14 group was clearly separated from that of the CON14 group, indicating a treatment-associated shift in gut microbiota composition.

### 3.8. Identification of Differentially Abundant Taxa and Enrichment Analysis

**Figure 8:**
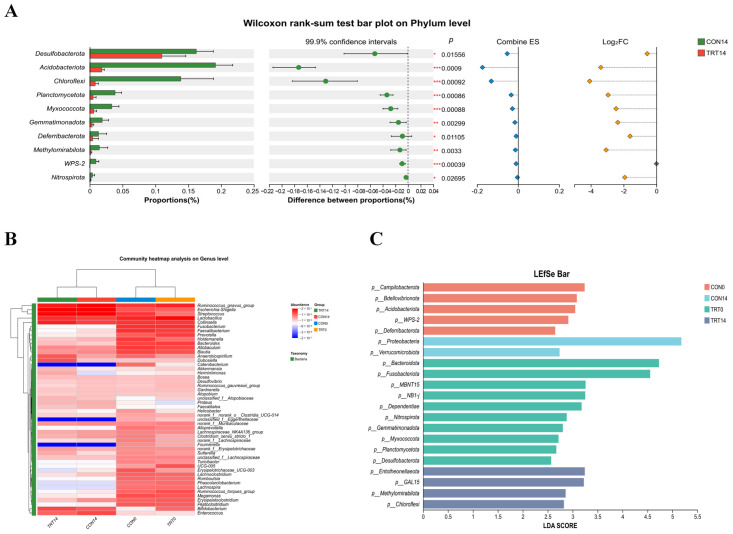
Identification of differentially abundant taxa and microbial biomarkers at different taxonomic levels. (**A**) Wilcoxon rank-sum test bar plot at the phylum level comparing CON14 and TRT14 groups; (**B**) Community heatmap analysis on genus level, the color depth represents the relative abundance of dominant genera; (**C**) Linear discriminant analysis effect size (LEfSe) bar chart showing the distribution of specific biomarkers. * *p* < 0.05, ** *p* < 0.01, *** *p* < 0.001.

Heatmap visualization and relative abundance bar plots at both the phylum and genus levels revealed treatment-associated differences in gut microbial composition (Figure 8A,B). Compared with the CON group, the relative abundances of Desulfobacterota, Acidobacteriota, and Chloroflexi were significantly lower in the TRT group (*p* < 0.05). In contrast, an increased relative abundance of bacteria commonly regarded as beneficial, such as members of the genus *Lactobacillus*, was observed in the TRT group. To further identify discriminative microbial features between groups, linear discriminant analysis effect size (LEfSe) was applied (Figure 8C). The analysis revealed distinct taxonomic signatures for each group. The CON14 group was characterized by enrichment of taxa such as *Entotheonellaeota*, whereas the TRT14 group exhibited a different microbial profile with specific enrichment of taxa belonging to the genus *Lactobacillus*. These taxa also showed higher relative abundance in the corresponding heatmap analysis, indicating consistency across analytical approaches. Collectively, these results demonstrate that PC treatment was associated with selective alterations in gut microbial composition, marked by differential enrichment of specific bacterial taxa during the later stage of intervention.

## 4. Discussion

In this study, an integrated multi-omics approach was used to investigate the protective effects of *Pulsatilla chinensis* (PC) in a canine model of antibiotic-associated diarrhea. LC–MS profiling identified anemoside B4, berberine, stigmasterol, and quercetin as the main bioactive constituents. Network pharmacology indicated that PC modulates 43 core targets across 13 signaling pathways, with hub genes including *AKT1*, *EGFR*, *IL6*, *TNF*, and *TP53*. Enrichment analysis highlighted the PI3K–Akt and IL-17 pathways as key regulatory axes. Molecular docking showed that stigmasterol and quercetin had strong binding affinities for *EGFR* and *AKT1*, and molecular dynamics simulations confirmed the stability of these interactions. In vivo, PC treatment attenuated diarrhea severity, reduced serum TNF-α and IL-6 levels, and increased IgG and IgA concentrations. 16S rRNA sequencing revealed that PC preserved gut microbial richness, enriched *Lactobacillus*, and reduced potentially pathogenic taxa such as *Escherichia–Shigella* and *Proteobacteria*. These findings suggest that PC exerts protective effects by modulating inflammatory responses and reshaping the gut microbiota, supporting its potential as a microbiota-modulating intervention for antibiotic-associated diarrhea in veterinary medicine [27]. Collectively, these results suggest that PC may exert protective effects by influencing inflammatory signaling, immunoglobulin production, and intestinal microbial ecology, providing a potential mechanistic rationale for its application in veterinary gastrointestinal disease. Recent advances in canine AAD research have highlighted the therapeutic potential of microbiome-targeted interventions. Shen et al. demonstrated that synbiotic supplementation (including chitosan oligosaccharides, *Bifidobacterium*, *Clostridium butyricum*, and *Lactiplantibacillus plantarum*) significantly reduced diarrhea severity, improved intestinal morphology, and upregulated tight junction protein expression in a canine AAD model [28]. Similarly, host-derived *Pediococcus acidilactici* GLP06 was shown to mitigate canine AAD by improving intestinal barrier function, regulating gut microbiota, and restoring metabolic homeostasis [29]. These findings underscore the growing recognition that restoration of microbial and mucosal homeostasis is critical for managing AAD in dogs, aligning with our observation that the protective effects of PC may involve the concurrent modulation of gut microbiota and host inflammatory responses.

The bioactive constituents of PC and their predicted molecular targets provide a mechanistic basis for the anti-inflammatory and epithelial-protective effects observed in PC-treated dogs. LC–MS profiling revealed multiple bioactive components in the PC extract, including anemoside B4, berberine, stigmasterol, and quercetin. In laying hens with *Clostridium perfringens*-induced necrotic enteritis, dietary supplementation with anemoside B4 (20 mg/kg) for 14 days attenuated intestinal morphological damage and increased the mRNA expression of claudin-1 and occludin in the jejunal mucosa [30]. Mechanistically, anemoside B4 has been shown to reprogram macrophage function by inhibiting pyruvate carboxylase, thereby alleviating colitis [31], and to suppress colitis-associated colon cancer progression via the Wnt/β-catenin signaling pathway [32]. In a mouse model of stress-induced diarrhea, oral administration of stigmasterol (40 mg/kg) for 7 days reduced fecal scores and attenuated colonic histopathological injury [33]. Consistent with these findings, network pharmacology analysis in the present study showed that the putative targets of PC were significantly enriched in pathways involved in inflammatory regulation and epithelial homeostasis, particularly the PI3K–Akt and IL-17 signaling pathways. Core targets such as *AKT1*, *IL6*, *TNF*, *TP53*, and *EGFR* occupied central positions within the protein–protein interaction network, suggesting that multiple constituents of PC may converge on key regulators of inflammation and tissue repair. The involvement of these targets in intestinal injury has been documented in other contexts. In dextran sulfate sodium (DSS)-induced colitic mice, inhibition of IL-6 and *TNF* signaling reduced mucosal inflammation and improved histological scores [34,35]. In a mouse model of colitis, activation of *EGFR* signaling by dietary ginkgetin (10 mg/kg) for 7 days reduced epithelial cell apoptosis and restored barrier function [36]. Molecular docking in this study further predicted that stigmasterol and quercetin, the two representative core components, might form stable interactions with AKT1 and EGFR, with binding energies below −9.0 kcal/mol. Furthermore, molecular dynamics simulations confirmed the stability of these most potent ligand-protein complexes, demonstrating that both stigmasterol and quercetin maintained stable interactions with EGFR, as evidenced by stable RMSD profiles over 100 ns simulations. Moreover, quercetin has been shown to mediate the PI3K/Akt/mTOR pathway, exerting antioxidant, anti-inflammatory, and anti-apoptotic effects in LPS-stimulated intestinal epithelial cell models [37]. These findings corroborate our molecular docking and dynamics simulation results, strengthening the mechanistic plausibility of PC’s anti-inflammatory effects through PI3K-AKT modulation. In summary, these integrated analyses indicate that the chemical composition of PC supports its potential to modulate inflammation and may contribute to epithelial homeostasis through multiple interacting targets and pathways, offering a mechanistic rationale for its protective effects in antibiotic-associated diarrhea.

PC treatment significantly reduced serum TNF-α and IL-6 concentrations in dogs with antibiotic-associated diarrhea on day 14, indicating attenuation of systemic inflammatory responses. These two cytokines are downstream effectors of the PI3K–Akt and NF-κB signaling pathways [38]. In a mouse model of 5-fluorouracil-induced intestinal mucositis, inhibition of the PI3K-Akt pathway reduced serum TNF-α and IL-6 levels and attenuated mucosal damage [39]. The cytokine reductions observed in the present study are consistent with these reports and suggest that PC treatment suppressed the inflammatory response triggered by antibiotic-induced intestinal disturbance. PC treatment also increased serum immunoglobulin concentrations. Compared with the CON group, dogs in the TRT group showed significantly higher IgG and IgA levels on day 14. IgA contributes to mucosal immune defense by limiting pathogen adhesion and maintaining microbial equilibrium at the epithelial interface, while IgG participates in systemic immune protection [40]. The concurrent elevation of IgG and IgA, alongside the decrease in IgM, suggests that PC treatment may facilitate a shift towards a more mature humoral profile following antibiotic-induced disturbance. These dynamic immunoglobulin shifts might also reflect alternative physiological processes. For instance, the changes could be driven by non-specific immune activation resulting from gut microbiota remodeling [41]. Alternatively, they may represent a secondary effect of accelerated mucosal healing, or reflect altered turnover rates of circulating immunoglobulins under conditions of reduced systemic inflammatory stress [42]. Collectively, PC administration reduced circulating pro-inflammatory cytokines and increased immunoglobulins in antibiotic-associated diarrheal dogs. These serum-level changes indicate suppression of inflammation and restoration of immune competence, providing physiological evidence for the host-directed effects of PC in this model.

The microbiota findings provide additional insight into ecological recovery associated with PC intervention. Antibiotic exposure resulted in marked depletion of microbial richness, as reflected by reduced Sobs and Chao1 indices, consistent with antibiotic-induced ecological disruption reported previously [43,44]. At the genus level, PC-treated dogs exhibited marked enrichment of *Lactobacillus* and reduced abundance of *Escherichia*–*Shigella* compared with the CON group on day 14. In weaned piglets challenged with enterotoxigenic *Escherichia coli*, dietary supplementation with *Lactobacillus plantarum* increased jejunal ZO-1 and occludin expression and reduced diarrhea incidence [45]. In mice with DSS-induced colitis, oral administration of *Lactobacillus rhamnosus* attenuated weight loss and reduced histologic injury scores [46]. Another study in broiler chickens infected with *Salmonella* showed that *Lactobacillus* supplementation increased the expression of barrier proteins such as ZO-1 and occludin, and improved the gut microbiota composition [47]. In human intestinal Caco-2 cells, co-incubation with *Lactobacillus* strains prevented TNF-α-induced barrier disruption and maintained transepithelial electrical resistance [48]. The enrichment of *Lactobacillus* observed in the present study is consistent with these reports and suggests that PC treatment promoted conditions favorable for potential barrier-supporting bacteria. Recent studies have further elucidated the mechanisms by which *Lactobacillus* species improve intestinal barrier function in AAD. A combination of *Lactiplantibacillus plantarum* ELF051 and Astragalus polysaccharides was shown to elevate the expression of tight junction proteins (ZO-1, occludin, claudin-1) via Smad signaling nodes, facilitating intestinal mucosal repair in AAD mice [49]. Conversely, the reduced abundance of *Escherichia*–*Shigella* and Proteobacteria in the PC group may reflect decreased inflammatory pressure, as expansion of these taxa has been linked to barrier dysfunction. In mice infected with attaching and effacing *E. coli*, increased *Escherichia* abundance correlated with reduced colonic claudin-3 expression and increased fluorescein isothiocyanate–dextran permeability [50]. Moreover, berberine, a primary bioactive constituent of PC, has been shown to alleviate enterotoxigenic *Escherichia coli*-induced mucosal barrier damage by suppressing pathogenic overgrowth and modulating the intestinal microbiome [51,52]. In the present study, PC administration was associated with directional shifts in gut microbiota, including enrichment of *Lactobacillus* and reduction in inflammation-associated genera. These compositional changes suggest a gut microbial profile that may be beneficial to intestinal barrier stability during recovery from antibiotic-associated diarrhea.

Reduced abundance of Desulfobacterota in PC-treated dogs is of particular relevance to the inflammatory and epithelial outcomes observed in this study. Members of this phylum are major producers of hydrogen sulfide, which at elevated concentrations disrupts mitochondrial respiration in colonocytes and increases epithelial permeability [53,54]. The concurrent reduction in Desulfobacterota and serum TNF-α/IL-6 in the TRT group suggests that PC-mediated suppression of this taxon may have contributed to the attenuation of mucosal inflammatory signaling, as hydrogen sulfide-driven epithelial injury is known to amplify pro-inflammatory cytokine release through NF-κB activation. Previous studies have shown that elevated Desulfobacterota abundance in patients with Crohn’s disease positively correlates with mucosal TNF-α levels and disease activity [55], a pattern directionally opposite to that observed in PC-treated dogs, further supporting the functional relevance of this taxon to the cytokine changes reported here. Decreased representation of Chloroflexi and Acidobacteriota in the TRT group is consistent with recovery from antibiotic-induced microbial disturbance, as these taxa have been associated with dysbiotic states and reduced colonization resistance in both animal models and clinical settings [56,57]. These shifts in microbial composition and diversity provide a microbiota-linked basis for the reduced inflammation and improved immunoglobulin profiles, supporting that gut microbiota remodeling contributes to the host-protective effects of PC in antibiotic-associated diarrhea.

Although the present model successfully induced diarrhea and gut microbiota dysbiosis following a 14-day course of enrofloxacin and metronidazole, it incompletely recapitulates naturally occurring AAD in veterinary practice. Clinical cases in veterinary practice involve diverse breeds, varying diets, and environmental exposures [58]. Most importantly, clinical patients receive antimicrobials for underlying primary infections, which can independently alter gut microbial ecology and host mucosal immunity [59]. Consequently, although this experimental model successfully validates the fundamental restorative mechanisms of PC against severe antimicrobial pressure, its real-world clinical efficacy may differ.

In silico approaches predicted interactions between PC constituents and key targets like *AKT1* and *EGFR*; however, these findings need future experimental validation. Since both groups received antibiotics, some benefits in the TRT group—such as reduced inflammation or microbiota shifts—may partly reflect spontaneous recovery rather than PC treatment alone. Future studies should include a healthy control group without antibiotics to better distinguish PC’s specific effects. Whether the PC-induced microbiota shifts directly caused the reduced inflammation and improved immunoglobulins, or merely occurred in parallel, remains unclear; fecal microbiota transplantation is needed to establish causality. Additionally, future studies should include direct histological or molecular assessment of the intestinal mucosa.

## 5. Conclusions

Integrated multi-omics analysis suggests that *Pulsatilla chinensis* alleviates canine antibiotic-associated diarrhea by potentially targeting the PI3K-Akt/IL-17 pathway via stigmasterol/quercetin–EGFR/AKT1 and remodeling the gut microbiota, reducing inflammation and enhancing humoral immunity. In vivo, PC treatment reduced serum pro-inflammatory cytokines including TNF-α and IL-6 and elevated immunoglobulins such as IgG and IgA, indicating attenuation of systemic inflammation and restoration of humoral immunity. Concurrently, PC reshaped the gut microbiota by enriching *Lactobacillus* and reducing inflammation-associated taxa including Desulfobacterota and *Escherichia–Shigella*. This coordinated host–microbiome action distinguishes PC from conventional anti-diarrheal agents that typically target only one aspect. From a clinical perspective, these findings support PC as a promising adjunctive therapy for AAD in veterinary practice. Future research should focus on validating the predicted molecular targets using biochemical assays, optimizing dosing regimens, and conducting long-term safety and efficacy trials in larger canine populations.

## Figures and Tables

**Table 1:** Main chemical components of PC extract.

No.	Compound Name	Formula	Adduct Ion	Precursor *m*/*z*	Reference *m*/*z*	RT (min)	Class
1	Anemoside B4	C_59_H_96_O_26_	[M − H]^−^	1220.3756	1219.6100	4.93	Triterpenoid Saponin
2	Anemoside A3	C_41_H_66_O_12_	[M + Na]^+^	773.4448	773.4400	4.92	Triterpenoid Saponin
3	Pulchinenoside A	C_41_H_66_O_12_	[M + H]^+^	751.4653	751.4615	3.98	Triterpenoid Saponin
4	Berberine	C_20_H_18_NO_4_	[M]^+^	336.1221	336.1230	4.14	Alkaloid
5	Quercetin	C_15_H_10_O_7_	[M − H]^−^	301.3820	301.0350	6.7	Flavonoid
6	Stigmasterol	C_29_H_48_O	[M + H]^+^	413.3783	413.3783	3.97	Phytosterol
7	Quercetin derivative	C_18_H_16_O_10_S	[M − H]^−^	423.0379	423.0392	3.38	Flavonoid
8	Cryptochlorogenic acid	C_16_H_18_O_9_	[M + H]^+^	355.1714	355.1027	3.33	Phenylpropanoid
9	Neochlorogenic acid	C_16_H_18_O_9_	[M − H]^−^	353.0874	353.0878	3.76	Phenylpropanoid
10	Caffeic acid hexoside	C_15_H_18_O_9_	[M − H]^−^	341.0884	341.0871	3.06	Phenylpropanoid
11	Isoferulic acid	C_10_H_10_O_4_	[M − H]^−^	194.0544	193.0506	4.22	Phenylpropanoid
12	Esculetin	C_9_H_6_O_4_	[M + H]^+^	179.0337	179.0340	3.51	Coumarin
13	Matairesinol	C_20_H_22_O_6_	[M − H]^−^	357.1352	357.1341	4.51	Lignan
14	Isocitric acid	C_6_H_8_O_7_	[M − H]^−^	191.0190	191.0197	0.84	Organic Acid
15	L-Malic acid	C_4_H_6_O_5_	[M − H]^−^	133.0134	133.0137	0.71	Organic Acid
16	Linoleic acid	C_18_H_32_O_2_	[M − H]^−^	279.2324	279.2331	5.87	Fatty Acid
17	2-Hydroxypalmitic Acid	C_16_H_32_O_3_	[M − H]^−^	271.2284	271.2279	6.5	Fatty Acid
18	L-Tryptophan	C_11_H_12_N_2_O_2_	[M − H]^−^	203.0818	203.0826	3.08	Amino Acid
19	Limonin	C_26_H_30_O_8_	[M + H]^+^	471.2015	471.201	4.85	Triterpenoid
20	Gluconic acid	C_6_H_12_O_7_	[M − H]^−^	195.0507	195.0508	0.64	Organic Acid

**Table 2:** Binding Energy Between Core Active Components of PC and Core Target Molecules.

Molecular Name	Binding Energy with *AKT1*	Binding Energy with *EGFR*	Binding Energy with *IL6*	Binding Energy with *MMP9*	Binding Energy with *TNF*	Binding Energy with *TP53*
Anemoside B4	−7.8	−7.5	−6.9	−6.5	−6.2	−7.1
Anemoside A3	−7.6	−7.2	−6.5	−6.1	−6.9	−6.0
Pulchinenoside A	−7.5	−7.4	−6.7	−8.3	−6.2	−7.9
Berberine	−8.2	−8.1	−7.4	−7.2	−7.8	−7.6
Stigmasterol	−9.4	−9.5	−8.4	−6.8	−6.9	−7.8
Quercetin	−9.3	−9.7	−8.1	−6.6	−7.0	−7.5

## Data Availability

Raw sequencing data are available in the NCBI Sequence Read Archive (accession no. PRJNA1091895). The remaining data presented in this study are available on request from the corresponding author. The data are not publicly available due to privacy restrictions.

## Abbreviations

The following abbreviations are used in this manuscript:

ANOVA	Analysis of variance
BP	Biological process
CC	Cellular component
CON	Control group
DDA	Data-dependent acquisition
ELISA	Enzyme-linked immunosorbent assay
FEL	Free energy landscape
GO	Gene Ontology
HESI	Heated electrospray ionization
IgA	Immunoglobulin A
IgG	Immunoglobulin G
IgM	Immunoglobulin M
IL-1β	Interleukin-1 beta
IL-6	Interleukin-6
KEGG	Kyoto Encyclopedia of Genes and Genomes
LC–MS	Liquid chromatography–mass spectrometry
LDA	Linear discriminant analysis
LEfSe	Linear discriminant analysis effect size
MD	Molecular dynamics
MF	Molecular function
NCE	Normalized collision energy
OTU	Operational taxonomic unit
PC	*Pulsatilla chinensis*
PCA	Principal component analysis
PCoA	Principal coordinate analysis
PPI	Protein–protein interaction
QC	Quality control
Rg	Radius of gyration
RMSD	Root mean square deviation
RMSF	Root mean square fluctuation
SD	Standard deviation
TIC	Total ion chromatogram
TNF-α	Tumor necrosis factor-alpha
TRT	Treatment group
UHPLC	Ultra-high-performance liquid chromatography
